# The Redox Balance in Erythrocytes, Plasma, and Periosteum of Patients with Titanium Fixation of the Jaw

**DOI:** 10.3389/fphys.2017.00386

**Published:** 2017-06-07

**Authors:** Jan Borys, Mateusz Maciejczyk, Adam J. Krȩtowski, Bozena Antonowicz, Wioletta Ratajczak-Wrona, Ewa Jabłońska, Piotr Załęski, Danuta Waszkiel, Jerzy R. Ładny, Piotr Żukowski, Anna Zalewska

**Affiliations:** ^1^Department of Maxillofacial and Plastic Surgery, Medical University of BialystokBialystok, Poland; ^2^Department of Physiology, Medical University of BialystokBialystok, Poland; ^3^Clinical Research Centre, Medical University of BialystokBialystok, Poland; ^4^Department of Oral Surgery, Medical University of BialystokBialystok, Poland; ^5^Department of Immunology, Medical University BialystokBialystok, Poland; ^6^Department of Conservative Dentistry, Medical University BialystokBialystok, Poland; ^7^Department of Emergency Medicine and Disaster, Medical University BialystokBialystok, Poland; ^8^Department of Restorative Dentistry, Croydon University HospitalCroydon, United Kingdom

**Keywords:** antioxidants, dentofacial deformities, miniplates and screws, oxidative stress, titanium fixation

## Abstract

Titanium miniplates and screws are commonly used for fixation of jaw fractured or osteotomies. Despite the opinion of their biocompatibility, in clinical practice symptoms of chronic inflammation around the fixation develop in some patients, even many years after the application of miniplates and screws. The cause of these complications is still an unanswered question. Taking into account that oxidative stress is one of the toxic action of titanium, we have evaluated the antioxidant barrier as well as oxidative stress in the erythrocytes, plasma and periosteum covering the titanium fixation of the jaw. The study group was composed of 32 patients aged 20–30 with inserted miniplates and screws. The antioxidant defense: catalase (CAT), glutathione peroxidase (GPx), superoxide dismutase-1 (SOD1), uric acid (UA), total antioxidant capacity (TAC), as well as oxidative damage products: advanced oxidation protein products (AOPP), advanced glycation end products (AGE), dityrosine, kynurenine, N-formylkynurenine, tryptophan, malondialdehyde (MDA), 4-hydroxynonenal (4-HNE), total oxidant status (TOS), and oxidative status index (OSI) were evaluated. SOD1 activity (↓37%), and tryptophan levels (↓34%) showed a significant decrease while AOPP (↑25%), TOS (↑80%) and OSI (↑101%) were significantly elevated in maxillary periosteum of patients who underwent bimaxillary osteotomies as compared to the control group. SOD-1 (↓55%), TAC (↓58.6%), AGE (↓60%) and N-formylkynurenine (↓34%) was statistically reduced while AOPP (↑38%), MDA (↑29%), 4-HNE (↑114%), TOS (↑99%), and OSI (↑381%) were significantly higher in the mandibular periosteum covering miniplates/screw compared with the control tissues. There were no correlations between antioxidants and oxidative stress markers in the periosteum of all patients and the blood. As exposure to the Ti6Al4V titanium alloy leads to disturbances of redox balance in the periosteum surrounding titanium implants of the maxilla and the mandible so antioxidant supplementation should be recommended to the patients undergoing treatment of dentofacial deformities with the use of titanium implants. The results we obtained may also indicate a need to improve the quality of titanium jaw fixations through increase of TiO_2_ passivation layer thickness or to develop new, the most highly biodegradable materials for their production.

## Introduction

Titanium and its alloys are commonly used for the production of medical implants. They have a broad range of applications in oral surgery and orthopedics as a bone fixation (plates, screws, rods, stabilizers, and wires), joint prostheses, dental implants and other devices used in reconstructive surgery (Borys et al., 2004). A common use of implants composed of titanium and its alloys results from good mechanical properties, resistant to corrosion and biocompatibility. Their higher biocompatibility in human tissues as compared to other metallic materials results from the presence of an inactive layer of titanium dioxide (TiO_2_) on the implant surface, which should reduce the corrosion potential of the metal (Tsaryk et al., 2007). Despite the opinion of their biocompatibility, in clinical practice symptoms of chronic inflammation around the fixation develop in some patients, even many years after the application of miniplates and screws (Peters et al., 2007; Olmedo et al., 2008; Lee et al., 2013). It is unknown what changes in the tissues surrounding the implant lead to these complications, however, it is believed that one of them may be an increased production of free radicals and reactive nitrogen species by exposure to titanium implants (Mentus, 2004; Peters et al., 2007; Tsaryk et al., 2007; Olmedo et al., 2008).

To date, it is still unclear whether titanium implants induce oxidative stress, which is a situation in which the antioxidant barrier is insufficient to quench the excess production of the reactive oxygen species (ROS). A failure to neutralize ROS leads to cellular metabolism disorders and oxidative damage to major biomolecules and cellular structures, including lipids, proteins, DNA and carbohydrates (Lushchak, 2014).

The aim of the study was to exam the influence of titanium implants on the antioxidant defense and oxidative stress in the serum, erythrocytes and periosteum covering jaw bone fixation locations in patients treated for dentofacial deformities.

## Materials and methods

### Patients

All procedures included in this study were approved by the Bioethics Committee of the Medical University of Bialystok (permission number R-I-002/3/2-16). After the explanation of the nature, purpose and potential risk of the study, a written informed consent was obtained from each patient.

The study and control patients were operated on at the Department of Maxillofacial and Plastic Surgery at the Medical University in Bialystok, Poland (from 28.01.2016 to 21.01.2017).

The study group was composed of 32 patients who had previously implants placed (21 women and 11 men aged 20–30, mean age–25 years, 8 months). In the study group, the osteotomy segments in the mandible were fixed with a 5-hole miniplate and 4 screws on the right and left side—in total 2 miniplates + 8 screws; while in the maxilla on the right and left side—with two 4-hole miniplates and 6 screws (in total 4 plates and 12 screws; MEDGAL Sp. z o.o., Bialystok, Poland). The Bioethics Committee gave us approval to carry out the research within 1 year only. We have not always been able to complete the tissue sampling in the allotted time period of 1 year, therefore the control and the study groups consist of different patients.

Jaw fixations were removed from 12 to 30 months after insertion of implants. The control group (C) consisted of 24 generally healthy patients (11 women and 13 men) aged 21–28 (mean age of 23 and 3 months), whose periosteum and blood were taken on the day of surgery, prior to insertion of titanium miniplates and screws. Both the control and study patients were operated on due to class III dentofacial deformities (underdevelopment of the maxilla and hypertrophy of the mandible).

The inclusion criteria for patients in the study group was the presence of maxillary bone fixations after surgical correction of dentofacial deformities. The inclusion criteria for patients in the study group and the healthy control were: absence of any former treatment for bone fractures or previous jaw osteotomies with the use of titanium fixations; a non-inflammation-induced healing process starting from the point of inserting fixations until their removal; age of patients 20–30. Patients and healthy controls had 18.5 ≤ BMI ≥ 24.5, were non- smokers and did not have any illnesses or a history of gastrointestinal disorders, hypertension, hyperlipidemia, liver or renal disease, diabetes, thyroid diseases, immunological disorders or other general diseases as well as periodontitis, gingivitis and active odontogenic infection foci. Patients and the control declared abstinence from alcohol or intoxicating drugs during the previous 2 months. The patients and the controls had meet the correct values of blood parameters (WBC 4.6–8.3 × 10^3^/μL, RBC 4.2–5.3 × 10^6^/μL, HGB 12.4–15.1 g/L, PLT 143-278 × 10^3^/μL; electrolytes level—sodium (Na 136.8–144.2 mmol/L) and potassium (K 3.7–4.8 mmol/L); Activated Partial Thromboplastin Time (APTT 24.3–34.1 sek), Prothrombin Time (PT 11.8–14.2 sek), International Normalized Ratio (INR 0.84–1.2), CRP (0.1–4.6 mg/L). For 1 month before the surgical procedure until the implant was removed, patients in the control and the study group had been on a diet containing 2,000 kcal including 55% carbohydrates, 15.5% of protein and 29.5% of fat. The diet was determined by a dietitian, and for the duration of the experiment the patients were left under his control. Exclusion criteria were: age below 20 and above 30; inflammatory complications and jaw synostosis disorders after operations on bimaxillary osteotomies; present or resolved systemic inflammation or within the oral cavity and coexistent systemic illnesses; treatment with antibiotics, corticosteroids during the previous 12 months; operations for other reasons in the year preceding the research; operation due to jaw fractures and/or subject to osteotomies of these bones in the past; addiction to alcohol and/or drugs. Patients taking antioxidants, vitamins, and dietary supplements were also excluded.

### Surgical procedure

All surgical procedures were performed by one qualified surgeon (JB). Jaw fixations (miniplates and screws) were removed under local anesthesia (2% lignocaine with noradrenalin) between the 12th and the 30th month (approximately 19 months on average). The research material consisted of a small fragments of periosteum (gray-pigmented) adhering to the titanium miniplate excised as a standard procedure during the removal of the maxillary bone fixations (Max1, Man1) or in the case of the control group- healthy periosteum taken separately from maxilla (Max C) and mandible (Man C) during bimaxillary osteotomy before implantation of the miniplates and screws. The gray- pigmented periosteum was aseptic (data not shown). Before the surgery in the fasting patients and controls, 10 mL of venous blood samples were collected in ethylenediaminetetracetic acid (EDTA) tubes and centrifuged 1500 × g at 4°C for 10 min to separate plasma and erythrocytes. Erythrocytes were washed three times in cold saline (0.9% NaCl) and hemolyzed by the addition of a nine-fold volume of cold phosphate buffer (50 mM, pH 7.4). In order to prevent sample oxidation and proteolysis, 10 μL 0.5 M BHT (butylated hydroxytoluene, BHT, Sigma-Aldrich, Germany) in acetonitrile and the protease inhibitor (1 tablet/10 mL of the buffer) (Complete Mini Roche, France) were added per 1 mL of plasma and erythrocytes and stored at −80°C in until assayed.

### Preparation of tissue homogenates

The tissues were removed, immediately frozen in liquid nitrogen and stored at −80°C until use. They were rinsed in ice-cold PBS (0.02 mol L^−1^, pH 7.0–7.2) to be cleaned from any remaining blood elements, weighted (laboratory weight KERN PLI 510-3M), placed in glass tubes, minced into small pieces, then diluted in ice-cold PBS (1:13) and homogenized with a homogenizer (Omni TH, Omni International, Kennesaw, GA, USA) on ice and sonicated with an ultrasonic cell disrupter (1800 J per sample, 20 s × 3 on ice, UP 400S; Hielscher, Teltow, Germany) for further cell membrane breakdown. To all samples the protease inhibitor (1 tablet/10 mL of the buffer) (Complete Mini Roche, France) as well as BHT (10 μL 0.5 M BHT in acetonitryle per 1 mL of the buffer; Sigma-Aldrich, Germany) were added. Homogenates were centrifuged for 10 min at 3,500 × g. The resulting supernatants were analyzed on the same day.

### Biochemical analysis

#### Determination of enzymatic and non-enzymatic antioxidants

Catalase (CAT, EC 1.11.1.6), glutathione peroxidase (GPx, EC 1.11.1.9), copper and zinc-containing superoxide dismutase 1 (SOD-1, E.C. 1.15.1.1) and protein were determined in the erythrocytes and tissue homogenates, uric acids (UA), total antioxidant capacity (TAC) and protein were analyzed in the plasma and tissue homogenates.

CAT activity was assessed colorimetrically measuring the decrease in absorbance at 240 nm as a consequence of hydrogen peroxide (H_2_O_2_) consumption (Aebi, 1984).

GPx activity was determined colorimetrically measuring the conversion of NADPH to NADP+ at 340 nm. One unit of GPx activity was defined as the amount of enzyme, which catalyzes the oxidation of 1 millimole NADPH/1 min (Paglia and Valentine, 1967).

SOD1 activity was estimated colorimetrically based on the ability of SOD to inhibit the autooxidation of epinephrine at pH 10.2. One unit of SOD activity was defined as the amount of enzyme, which inhibits epinephrine oxidation by 50% (Misra and Fridovich, 1972).

UA was measured colorimetrically using a commercial kit QuantiChromTM Uric Acid Assay Kit DIUA-250 (BioAssay Systems, Harward, CA, USA). This method is based on the formation of a blue complex with iron, which is determined at a wavelength 490 nm.

The concentration of TAC was estimated in triplicate samples by 2,2′-azinobis(3-ethylbenzo-thiazoline-6-sulfonate) (ABTS^·+^)-based colorimetric method described by (Erel, 2004). Changes in absorbance of the reaction solution was measured at 660 nm and the results were expressed as μmol Trolox equivalent/100 of the total protein.

All assays were performed in a duplicate samples, except for the TAC determination (see above) and converted to mg of the total protein.

#### Determination of oxidative stress markers

Oxidative modifications products were assessed both in the plasma and tissue homogenates.

Advanced Oxidation Protein Products (AOPP) were estimated colorimetrically using a method Kalousová et al. (2002), which measures the total iodide ion oxidizing capacity of the samples. Absorbance at 340 nm was measured immediately by Infinite M200 PRO Multimode Microplate Reader, Tecan.

Advanced glycation end products (AGE) were estimated spectrofluorimetrically at the excitation and emission wavelengths of 350 and 440 nm using Infinite M200 PRO Multimode Microplate Reader, Tecan. Results were expressed as fluorescence/mg of the total protein.

The content of dityrosine, kynurenine, N-formylkynurenine and tryptophan was analyzed spectrofluorimetrically on 96-well microplates measuring the characteristic fluorescence at 330/415, 365/480, 325/434, and 95/340 nm respectively by Infinite M200 PRO Multimode Microplate Reader, Tecan. Results were expressed as fluorescence/mg of the total protein.

Lipid peroxidation was estimated colorimetrically using the Thiobarbituric Acid Reactive Substances (TBARS) method for measuring a malondialdehyde (MDA). 1,3,3,3 tetraethoxypropane was used as a standard (Buege and Aust, 1978).

The concentration of 4-hydroxynonenal (4-HNE) protein adducts was measured by commercial enzyme-linked immunosorbent assay (ELISA) according to the manufacturer's instructions (OxiSelect™ HNE Adduct Competitive ELISA Kit, Cell Biolabs, Inc. San Diego, CA, USA). The quantity of 4-HNE protein adducts was determined colorimetrically from a calibration curve for 4-HNE-BSA.

Total oxidant status (TOS) was measured colorimetrically based on the oxidation of ferrous ion (Fe^2+^) to ferric ion (Fe^3+^) in the presence of oxidants comprised in a sample (Erel, 2005). Changes in absorbance of the reaction solution were measured bichromatically (560/800 nm) in triplicate samples. The results were expressed as micromolar hydrogen peroxide (H_2_O_2_) equivalent per mg of the total protein (μmol H_2_O_2_ Equiv/mg of the total protein).

Oxidative stress index (OSI) was calculated according to the formula: OSI = TOS/TAC·100% (Knaś et al., 2016).

The total protein content was determined colorimetrically using the bicinchoninic acid assay (BCA assay) with bovine serum albumin (BSA) as a standard (Thermo Scientific PIERCE BCA Protein Assay Kit, Rockford, IL, USA).

**Figure 1:**
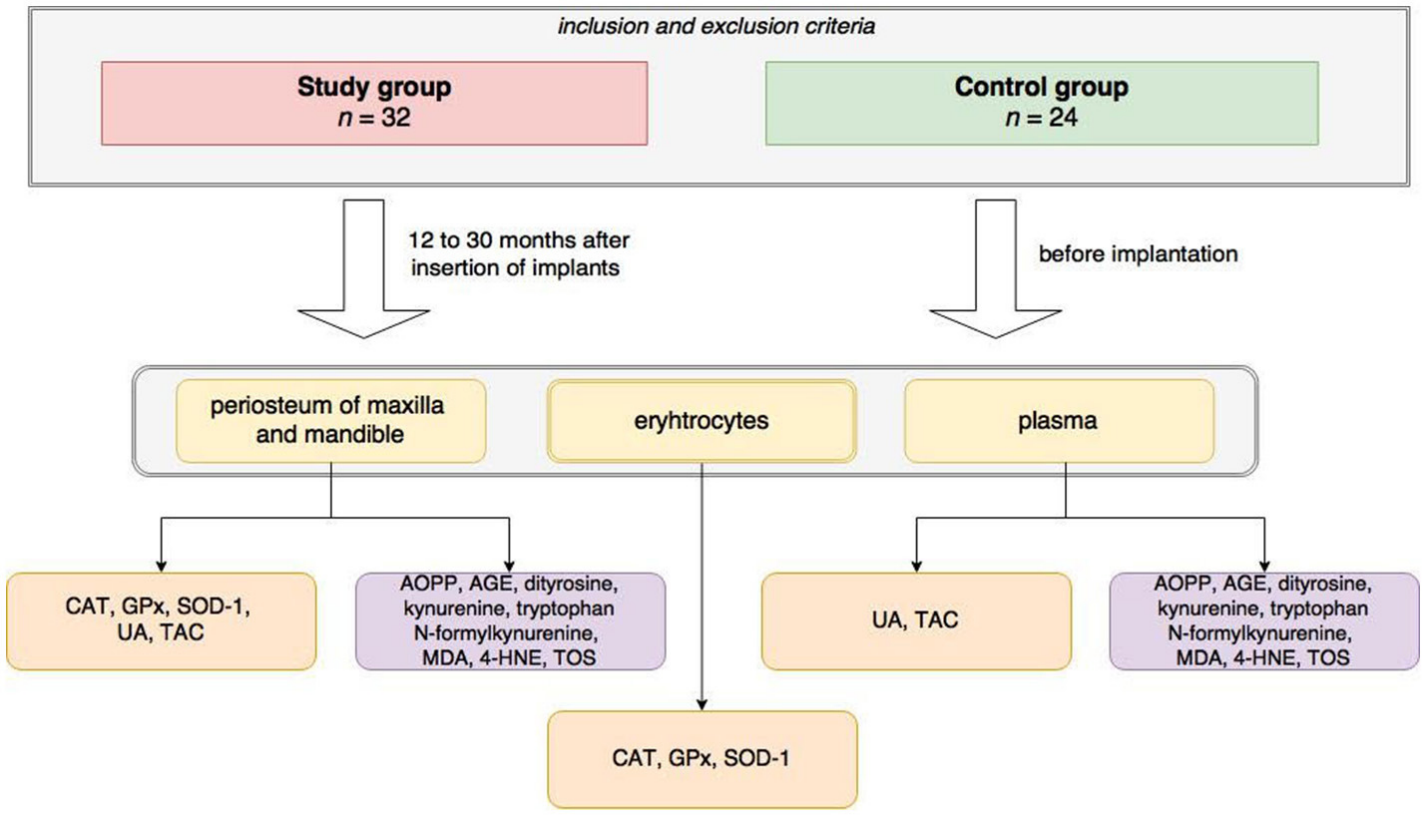
Graphical representation of the experiment scheme. In the research material (periosteum of maxilla and mandible, erythrocytes and plasma) in the study and control group were evaluated: activity/concentrations of some antioxidants (SOD-1, superoxide dismutase-1; GPx, glutathione peroxidase; CAT, catalase; UA, uric acid; TAC, total antioxidant capacity) as well as oxidative modification products (AGE, advanced glycation end products; AOPP, advanced oxidation protein products, MDA, malondialdehyde, TOC, total oxidant capacity).

All assays were performed in duplicate samples, except for the TOS determination (see above) and converted to mg of the total protein. Graphical representation of the experiment was presented on Figure 1.

### Statistical analysis

The data were reported as median, minimum and maximum. All analyses were performed using Statistica 12.0 (Statsoft, Cracow, Poland). The Kolmogorov-Smirnov test showed no normal distribution of the obtained results, which was the reason for using nonparametric methods. The control and Max1, Man1 groups were compared using the non-parametrical U Mann-Whitney test. The associations between the antioxidants, oxidative stress markers in the tissue homogenates and plasma antioxidants and oxidative stress markers concentrations as well as protein concentrations and the time elapsed since the surgery and removal of the miniplates/screw were analyzed using the Spearman Correlation Coefficient. Differences with *p* ≤ 0.05 were considered significant.

## Results

### Maxilla

**Figure 2:**
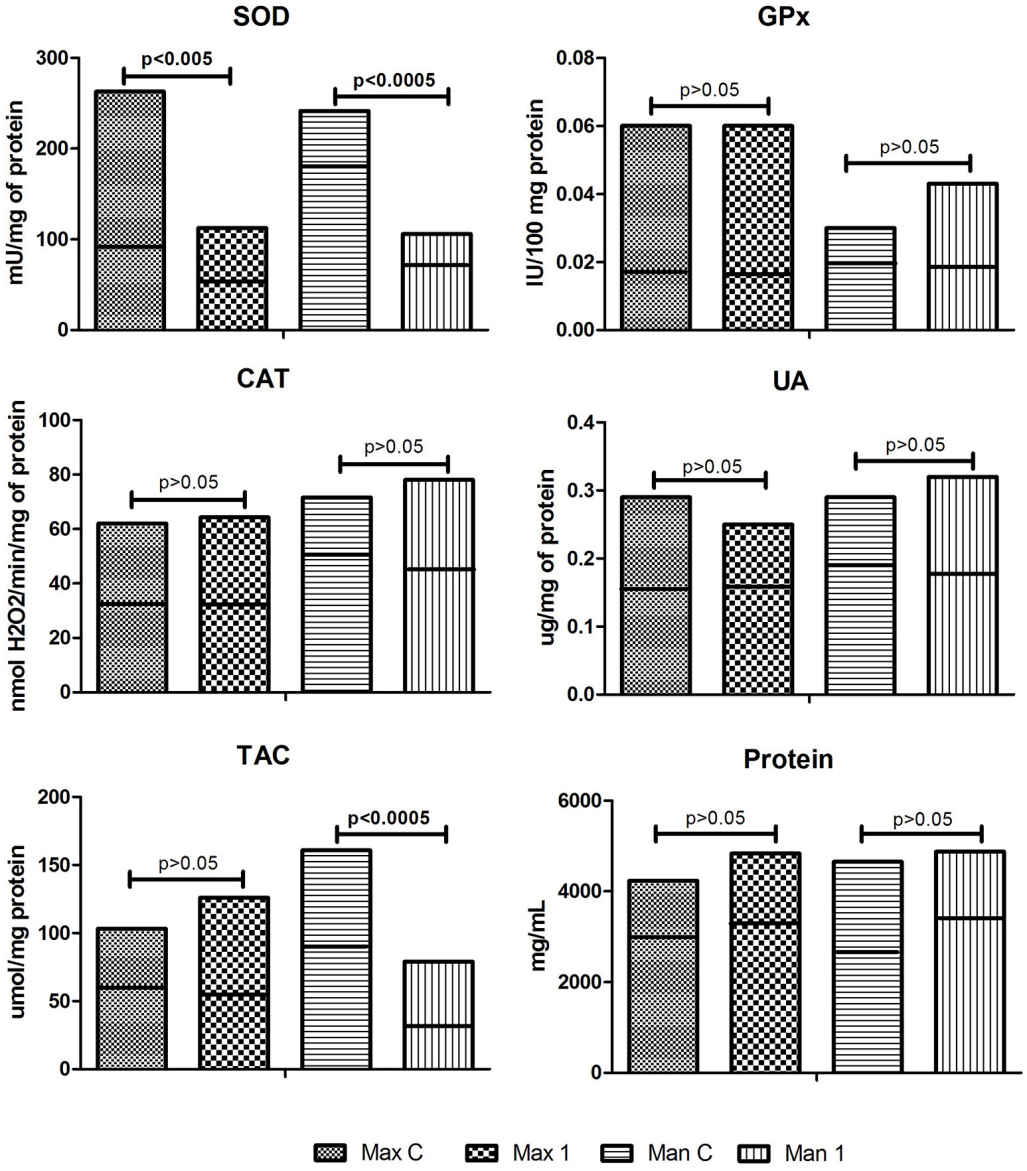
Enzymatic and non-enzymatic antioxidants and protein concentration in the control and examined tissue homogenates. Max C, maxilla control; Man C, mandibula controll; Max 1, maxilla of the study group; Man 1, mandibula of the study group; SOD, superoxide dismutase, GPx, glutathione peroxidase, CAT, catalase, UA, uric acid, TAC, total antioxidant capacity. Horizontal line on the chart indicated the median.

The activity of SOD1 (↓37%) was significantly decreased in tissue homogenates (maxillary gray- pigmented periosteum covered miniplates or screws) of patients who undergo bimaxillary osteotomies compared to the control periosteum (*p* = 0.0003). The activity of CAT and GPx and concentrations of TAC, UA as well as protein concentration did not differ between gray- pigmented periosteum of Max1 and the periosteum of the maxilla of the control group (Figure 2).

**Figure 3:**
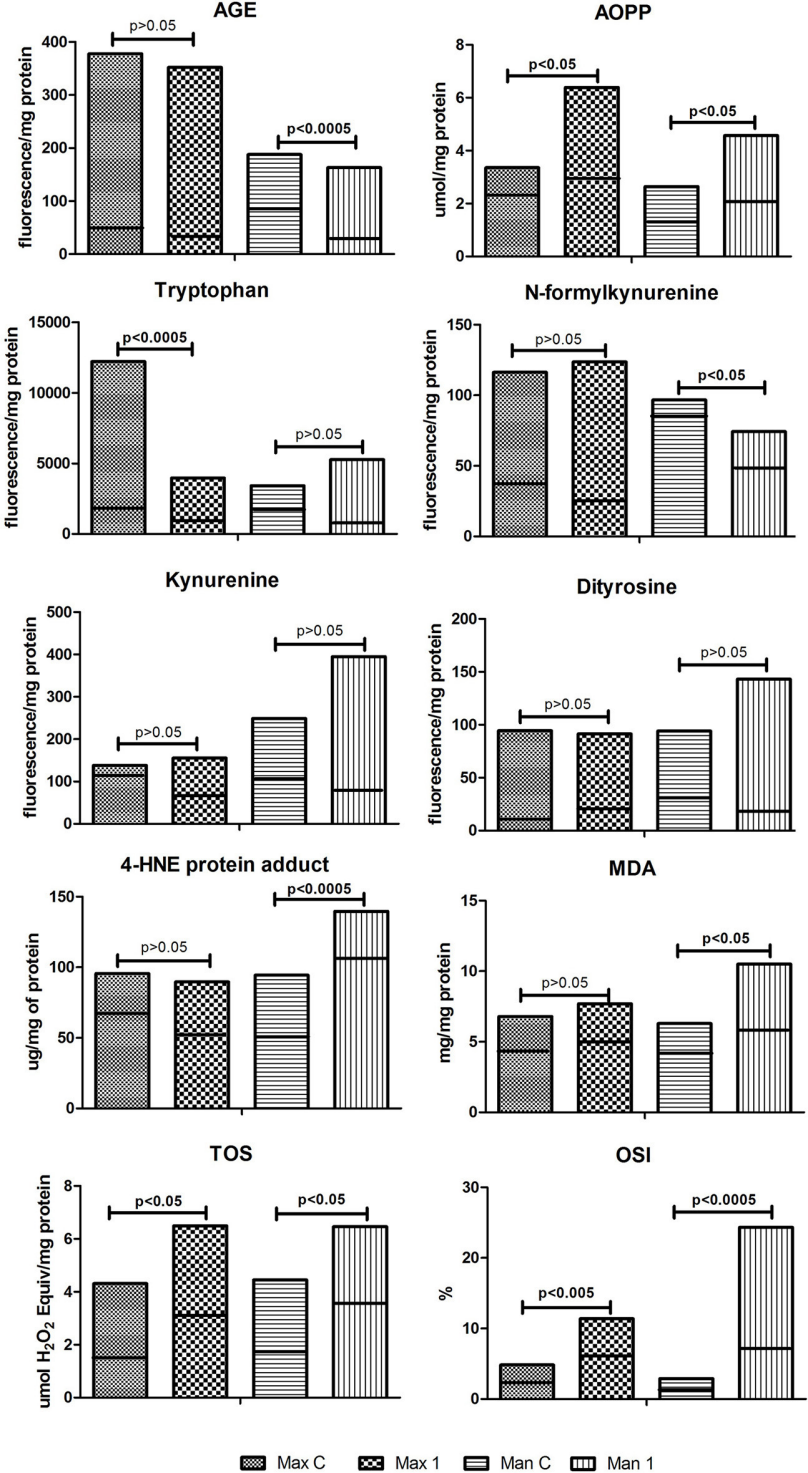
Oxidative modification products and markers of oxidative stress in the control and examined tissue homogenates. Max C, maxilla control; Man C, mandibula control; Max 1, maxilla of the study group; Man 1, mandibula of the study group; AGE, advanced glycation end products; AOPP, advanced oxidation protein products; MDA, malondialdehyde; TOC, total oxidant capacity; OSI, oxidative status index. Horizontal line on the chart indicated the median.

Regarding oxidative stress markers, only the concentration of tryptophan were decreased in the gray- pigmented periosteum of Max1 group compared to the periosteum of the maxilla of control (↓34%, *p* = 0.006). The concentration of AOPP (↑25%), TOS (↑80%) as well as OSI (↑98%) were significantly elevated in homogenates of maxillary periosteum taken from region of miniplates/screw as compared to the periosteum of the maxilla of the control (*p* = 0.035, *p* = 0.01, and *p* = 0.001, respectively). All remaining parameters of oxidative stress were similar in periosteum of Max1 and the control patients (Figure 3).

### Mandibula

As presented in Figure 2, with the exception of SOD1 and TAC, most of the examined parameters of antioxidant barrier, showed similar pattern in homogenates of mandibular periosteum covered miniplates/screw compared with the control tissue. Man1: GPx activity, UA, and protein concentration were comparable with the results of the control. SOD1 activity (↓55%) and TAC concentration (↓58.6%) in the homogenates of mandibular periosteum were significantly downregulated as compared to the control (*p* = 0.0009 and *p* = 0.0007, respectively).

Man1 group significantly decreased concentration of AGE (↓60%) and N-formylkynurenine (34%) when compared to the control group (*p* = 0.008 and *p* = 0.04, respectively). AOPP(↑38%), MDA (↑29%), 4-HNE protein adduct (↑114%), TOS (↑99%) concentrations as well as OSI (↑250%) were significantly elevated in the periosteum of Man1 patients as compared to the control group (*p* = 0.03, *p* = 0.04, *p* = 0.0002, *p* = 0.0005, *p* = 0.0005) (Figure 3).

Both Man1 and control groups showed similar tryptophan, kynurenine, ditirosine concentrations in their periosteum (Figure 3).

### Blood antioxidants barrier and oxidative stress markers

As presented in Tables 1, 2, with the exception of plasma AOPP in the study group, most of the examined parameters of antioxidant barrier and oxidative stress markers, showed a similar pattern in blood pellets and plasma of the control and experimental groups. We observed a significant decrease in plasma AOPP concentrations (*p* = 0.01) as compared to the control (Table 2).

**Table 1:** Enzymatic, non-enzymatic antioxidants and protein concentration in blood cells or plasma of the control and examined patients.

	**C *n* = 24, M(min–max)**	**Study group *n* = 32, M(min–max)**
SOD mU/mg of protein	145.982 (102.037–209.085)	154.859 (98.854–211.821)
GPx IU/100 mg protein	0.013(0.011–0.018)	0.013(0.010–0.016)
CAT nmol H_2_O_2_/min/mg of protein	7.765(2.144–14.875)	6.951(1.095–17.758)
Plasma UA (μg/mg of protein)	0.169(0.071–0.256)	0.162(0.050–0.222)
Plasma TAC μmol/mg protein	79.97(63–109.06)	79.71(66.3–109.28)
Protein mg/mL	4,570.750 (3,576.900–5,514.300)	4,662.400 (3,596.800–5,317.600)

*SOD, superoxide dismutase; GPx, glutathione peroxidase; CAT, catalase; UA, uric acid; TAC, total antioxidant capacity; BC, M (min-max), median (minimum-maximum); C, control group*.

**Table 2:** Oxidative modification products and markers of oxidative stress in the plasma of the control and examined patients.

	**C *n* = 24, M(min–max)**	**Study group *n =* 32, M(min–max)**
AGE fluorescence/mg protein	493.629 (311.700–690.682)	536.300 (324.161–734.959)
AOPP umol/mg protein	0.399(0.223–0.722)	0.356(0.185–0.546) ↓^x^
Tryptofan fluorescence/mg protein	6,753.227 (5,412.820–8,678.744)	6,436.073 (5,063.058–8,234.360)
N-formylkynurenine fluorescence/mg protein	386.517 (239.529–582.396)	438.406 (205.043–644.803)
Kynurenine fluorescence/mg protein	697.819 (504.000–932.511)	690.890 (354.315–974.361)
Dityrosine fluorescence/mg protein	564.164 (357.300–754.105)	553.884 (285.143–794.734)
4-HNE protein adduct μg/mg protein	26.51(19.7–56.23)	28.51(27.17–46.03)
MDA mg/mg protein	4.836(2.559–9.270)	5.430(2.272–9.823)
TOC μmol H_2_O_2_ Equiv/mg protein	0.08(0.005–1.054)	0.079(0.01–0.57)
OSI %	0.093(0.006–1.2)	0.092(0.016–0.53)

*AGE, advanced glycation end products; AOPP, advanced oxidation protein products; MDA, malondialdehyde; TOC, total oxidant capacity; OSI, oxidative status index; M (min–max), median (minimum-maximum); C, control group*.

↓x *- significant decrease, with p < 0.05 between C and study group*.

### Correlations

There were no correlations between antioxidants and oxidative stress markers in periosteum of Max1, Man1, and the plasma. There was no correlation between antioxidants and oxidative stress markers in periosteum of Max1, Man1, and age and BMI index of the patients. There were no correlations between antioxidants and oxidative stress markers in periosteum of Max1, Man1, and the time elapsed since the surgery and removal of the miniplates/screws. There was correlation between tryptophan and protein concentrations in periosteum of Max 1 group (*p* = 0.01, *r* = −0.56).

## Discussion

In the present study we showed that exposure to the Ti6Al4V titanium alloy leads to disrupted redox balance in the periosteum surrounding titanium implants of the maxilla and the mandible, however greater weakening of antioxidant systems, ROS generation and greater intensity of oxidative modifications were observed in the mandibular periosteum as opposed to the maxillary periosteum. We also proved that oxidative damage to the periosteum of the maxilla and mandible exposed to Ti6Al4V does not depend on the time elapsed since the surgery and removal of the miniplates/screw as well as that chronic exposition of the periosteum of the maxilla and mandible to Ti6Al4V does not influence the general redox balance.

The surface of titanium implants for numerous applications (joints prosthesis, bone fixation) is covered with a layer of titanium dioxide (titanium dioxide layer), which is responsible for the biocompatibility of the implant and reduces the corrosion potential of the metal (corrosion resistance and biocompatibility) (Tengvall et al., 1989; Tsaryk et al., 2007). However, in the human body, as a result of mechanical friction (metal on tissues, metal on metal) and electrochemical phenomena, the TiO_2_ gets damaged, which leads to corrosion processes and the accumulation of wear debris in the region of implantation, plasma (Hallab et al., 2000), as well as in distant organs: lymph nodes, the spleen and liver (Case et al., 1994; Hallab et al., 2000; Urban et al., 2000). Titanium release is a result of the anodic corrosion process, taking place in the TiO_2_ layer. The consequence of the accumulation of titanium ions is their phagocytosis by macrophages. Phagocytosis is linked to stimulation of respiratory processes and the formation of reactive oxygen species by activation of NADPH oxidase (Federico et al., 2007). Generated large quantities of superoxide anion ${\text{O}}_{2}^{\cdot -}$ enhance the secretion and the activity of IL6 (interleukin 6) as well as monocyte chemoattractant protein (MCP-1 protein), which promotes the infiltration of monocytes and subsequent ROS formation (Sung et al., 2002). Moreover, the cathodic part of the corrosion of titanium implants, at physiological pH, reduces oxygen to hydrogen peroxide, which indeed is not a free radical, but has oxidizing ability of both proteins and lipids (Haliwell et al., 2000). Hydrogen peroxide may react with the TiO_2_-layer producing highly reactive and the most dangerous reactive oxygen species- hydroxyl radical (Lee et al., 2005). Evidence showed that an increase in ROS formation may generate a situation in which the processes of bone formation are disturbed, and the processes responsible for bone resorption are enhanced. This also result in osteolysis around the implants, and consequently lead to an aseptic loss of the implant. However, it is still not known whether oxidative stress is induced under conditions of prolonged exposure to titanium (Hallab et al., 2000; Kinov et al., 2006; Tsaryk et al., 2007) in the periosteum of the mandible and the maxilla.

Among 32 patients undergoing double-stranded osteotomy, 18 had decided to remove jaw joints mainly due to the desire to get rid of non-functional implants, which could in the future be the cause of artifacts that would hamper proper assessment of CT images, MRI, and the risk of foreign body reactions. Another reason for the removal of miniplates and screws was discomfort connected with palpably felt fixations (6 patients), increased cold sensitivity (4 patients) and planned implant treatment of lost teeth (4 patients). During removal of the fixations the presence of gray- pigmented periosteum covering the miniplates and screws (Supplementary Image 1) was observed. Our clinical observations and literature data on the impact of metallic products on the surrounding tissues prompted us to evaluate the redox balance parameters in tissues surrounding titanic jaw fixtures.

It was documented that under a ROS increase, the level/activity of antioxidants may be increased, decreased or unchanged, which depends on the efficiency of a given tissue in fighting ROS and the level of ROS generation. Thus, both situations in which the increase in ROS generation (↑ TOS- 99% mandible, 80% maxilla) is accompanied by: a significant reduction in TAC concentrations, with unchanged concentrations/activity of other antioxidants (except for reduced activity of SOD 1) in the periosteum of the mandible as well as a lack of observed changes (except for reduced activity of SOD 1) of the antioxidative barrier of the periosteum of the maxilla exposed to titanium ions are equivalent to a shifted oxidative/antioxidative balance in the gray- pigmented periosteum of both the mandible and maxilla toward the oxidative status. However the antioxidant system of the mandible seems to be more affected.

A significant reduction of SOD1 activity in periosteum of Max1 and Man1 vs. appropriate controls, is in agreement with the results of others (Case et al., 1994; Hallab et al., 2000; Urban et al., 2000; Tsaryk et al., 2007; Saquib et al., 2012; Lee et al., 2013) and could have serious consequences for the process of bone healing. The non-neutralized superoxide anion could bind to the nitric oxide (NO) (Reynolds et al., 2017), harming its action on endothelial cells, which could have a negative effect on the neoangiogenesis process, and thus differentiation of osteopregenitor cells in osteoblasts at the fixation site.

The weakening of antioxidant response of the cell/tissue leads to oxidative modification of cellular components and it is evidenced by enhanced level of oxidatively modified cellular constituents or increased OSI (Sen et al., 2014; Knaś et al., 2016; Kołodziej et al., 2017; Maciejczyk et al., 2017).

Our study showed that both gray-pigmented periosteum of the maxilla and mandible exhibit greater susceptibility of their cellular elements to oxidative damage when exposed to titanium oxide *vs*. appropriate controls, regardless of the time elapsed between the insertion of the implant and its removal. It should be noticed, however, that greater diversity and intensity of oxidative modifications were observed in the periosteum of the mandible than in the periosteum of the maxilla. Our results showed a significant increase only in AOPP (↑25%) concentrations in the gray-pigmented periosteum of the maxilla as compared to the control, whereas in gray- pigmented periosteum of the mandible, we observed a significant increase in AOPP (38%) and in MDA (29%) and 4-HNE protein adduct (114%) as compared to the periosteum of the mandible collected before insertion of titanium implants. The intensity of oxidative stress determined by the OSI also confirmed that the periosteum covering the implants in the mandible (↑OSI 250%) is more exposed than in the maxilla (↑OSI 98%) to an oxidant attack generated if exposed to the Ti6Al4V titanium alloy. The observed inability to respond effectively to ROS input could have a negative effect on bone healing. One important factor of bone healing process is the differentiation of osteoprogenitor cells into osteoblasts and synthesis of organic substance, i.e., mostly collagen and the components of ground substance of the organic stroma of the bone (Borys et al., 2004). ROS reactions with proteins lead to irreversible changes in the structure of oxidized proteins as well as to a loss of their biological function. In the aspect of bone healing, this may result in the formation of an incorrectly built organic part of the bone tissue with poor mechanical resistance. What is more, Sheikhi et al. (2001) provide evidence that oxidized fatty acids stimulate adipogenesis and suppress osteoblastogenesis and directly stimulate formation as well as activity of osteoclasts (Sheikhi et al., 2001). It is very likely that intensive oxidative protein modification, expressed by elevated AOPP and also a greater number of lipid peroxidation products may interfere with the metabolism of the organic part of bone tissue and cause apertures of the mandible fracture that we observed in x-ray images of some patients (data not published) 14–18 months after the implantation.

This experiment does not explain the difference in the antioxidative response and susceptibility to oxidative damage. In our research, we have confirmed that the gray color of the periosteum was caused by titanium ion incrustation, what is more, we have observed that titanium ion concentrations were significantly higher in the mandible than in the maxilla (work in review). The explanation of these observations exceeds the scope of current work and is not explained in the available literature. We can only assume that the latter result is the result of different “motor function” of both analyzed bones. Among bones of the facial part of the skull, the mandible is the only moveable bone whose movement is controlled by strong muscles. Micromovements of the fixed osteotomy fragments caused by mandible movement may increase the phenomenon of friction (fretting) between the screws and the miniplate (metal on metal corrosion), which increases the corrosion of titanium elements and the release of titanium ions into the periosteum surrounding the implant and contributes to higher ROS generation, which in the situation of observed inefficiency of antioxidative systems results in greater intensity of oxidative damage as compared to the maxilla. We anticipate that antioxidant supplementation would be helpful in this group of patients, which could compensate for oxidant/antioxidant disturbances at the implantation site, and thus prevent cell damage by oxidative processes.

Kynurenic acid and N-formylkynurenine belong to the primary biologically- active tryptophan metabolites that are formed during enzymatic reactions in the kynurenine pathway. The results of our research reveal that the kynurenine pathway is not activated in the process of bone healing in the case of titanium implant fixation, which is advantageous from the point of view of the bone healing process. Tryptophan degradation products in the kynurenine pathway inhibit osteoprogenitor cell differentiation, which results in inhibition proliferation and differentiation of osteoblasts and impairs bone healing (Sas et al., 2007). Apart from reduced concentrations of N-formylkynurenine in the mandibular periosteum, which probably results from oxidative modifications of 2,3-dioxygenase or indoleamine 2,3-dioxygenase, which convert tryptophan to N-formylkynurenine and reduced tryptophan concentrations in the maxillary periosteum, we did not observe any significant changes in the kynurenine pathway. The negative correlation between the tryptophan content and protein concentrations in homogenate of the gray- pigmented periosteum of the maxilla may suggest the use of tryptophan in the process of the protein part formation in the bone tissue remodeling process.

The absence of determinations of titanium concentrations in the blood does not allow us to conclude, whether it is released into the human blood system as it was observed in the experimental models (Olmedo et al., 2008; El-Shenawy et al., 2012). However, we could claim that chronic exposure to titanium implants in the jaw did not cause the general redox disturbances, which is obviously an advantageous phenomenon considering the general health aspect.

While analyzing the results obtained, one needs to consider the limited panel of analyses used in the experiment. Determination of other antioxidants or oxidative stress markers or the use of other analytical methods may result in obtaining different results and conclusions. It should be also underlined that MDA was measured via its reaction with tiobarbituric acid (TBA), which is not specific for lipids only and many amino acids, carbohydrates and aldehydes may react with TBA under the assay conditions. A weakness of this study is also the small size of the groups; however, it should be emphasized that that the experiment included all patients meeting the inclusion criteria reporting operative treatment for class III dentofacial deformities or to continue this treatment (removal of jaw fixations). The fact that our research qualified patients within the same age group—20–30 years can be regarded as a strength of the experiment. At this stage of life, a human organism reaches its peak bone mass, and the processes of build-up and resorption of bone tissue are balanced. It should be also underlined that in a clinical trial in a significant majority of the treated patients redox balance disorders in the periosteum surrounding titanium implants in the jaw are difficult to detect with currently available non-invasive diagnostic methods (clinical and biochemical blood tests, conventional radiological picture–Supplementary Image 2) due to their subclinical nature. However, our results indicate a persistence of oxidative stress in patients around the jaw bone fixation regardless of the time elapsed since the operation face skeletal defects. Aseptic chronic inflammation resulting primarily in response to the consumption of the products introduced into the body of metallic bodies, may cause osteolysis around the implant process and lead to a loss e.g., prosthetic joints or bone fractures.

## Conclusions

1. Exposure to the Ti6Al4V titanium alloy leads to oxidant/antioxidant disturbances as well as oxidative damage to the periosteum surrounding the titanium implants of the maxilla and the mandible, although no clinical signs are observed.
2. Antioxidants supplementation (e.g., specific diet: green tea, aronia and blueberries juice, cocoa, curcumin, nuts, tomato, red vine etc. or spirulin, vitamin E, C supplementation) should be recommended to the patients undergoing treatment of skeletal defects with the use of titanium implants, as it could alleviate and/or prevent the damaging effects of oxidative stress in periosteum surrounding the implants.
3. The obtained results suggest sustained presence of oxidative stress among patients in the area of jaw bones fixations, independently of time, that has passed from the surgery of dentofacial deformities. It may indicate a need to improve the quality of used jaw bone fixations through increase of TiO_2_ passivation layer thickness in miniplates and screws in the process of hard anodizing or a need to search for new materials to produce fixations, preferably biodegradable in tissues of human organism. However, this requires further research.

## Author contributions

We declare that the paper “Antioxidant response and oxidative stress in patients with titanium fixation of the jaws JB, MM, AK, BA, WR, PZa, DW, JŁ, and AZ has not been published before. The paper is not under consideration for publication anywhere else and it was read and approved by all co- authors. All authors agree to the submission of the manuscript to the Frontiers in Physiology. JB: conceptualized, collection of the material, interpreted of data, wrote of the manuscript. MM: conceptualized, did laboratory determination, did performance of the graphic part of the manuscript. AK: conceptualized, interpreted of data, final approval of the version to be published. BA: conceptualized, did literature survey. WR: did statistical analysis, EJ: final approval of the version to be published. DW: did literature survey, final approval of the version to be published. PZa: collection of the material. JŁ: did literature survey, final approval of the version to be published. PŻu: final approval of the version to be published, English correction. AZ: conceptualized, did laboratory determination, interpreted of data, wrote of the manuscript.

### Conflict of interest statement

The authors declare that the research was conducted in the absence of any commercial or financial relationships that could be construed as a potential conflict of interest.

## Supplementary material

Supplementary Image 1 The gray- pigmented periosteum.

Supplementary Image 2 X ray picture of the patients 23 months after jaw fixation (Siemens Aristos VB20D, 55kV).
