# Supplementary figures and images for: The Redox Balance in Erythrocytes, Plasma, and Periosteum of Patients with Titanium Fixation of the Jaw
Source: Front Physiol. 2017 Jun 7;8:386. doi: 10.3389/fphys.2017.00386 (PMC5461302; doi:10.3389/fphys.2017.00386)

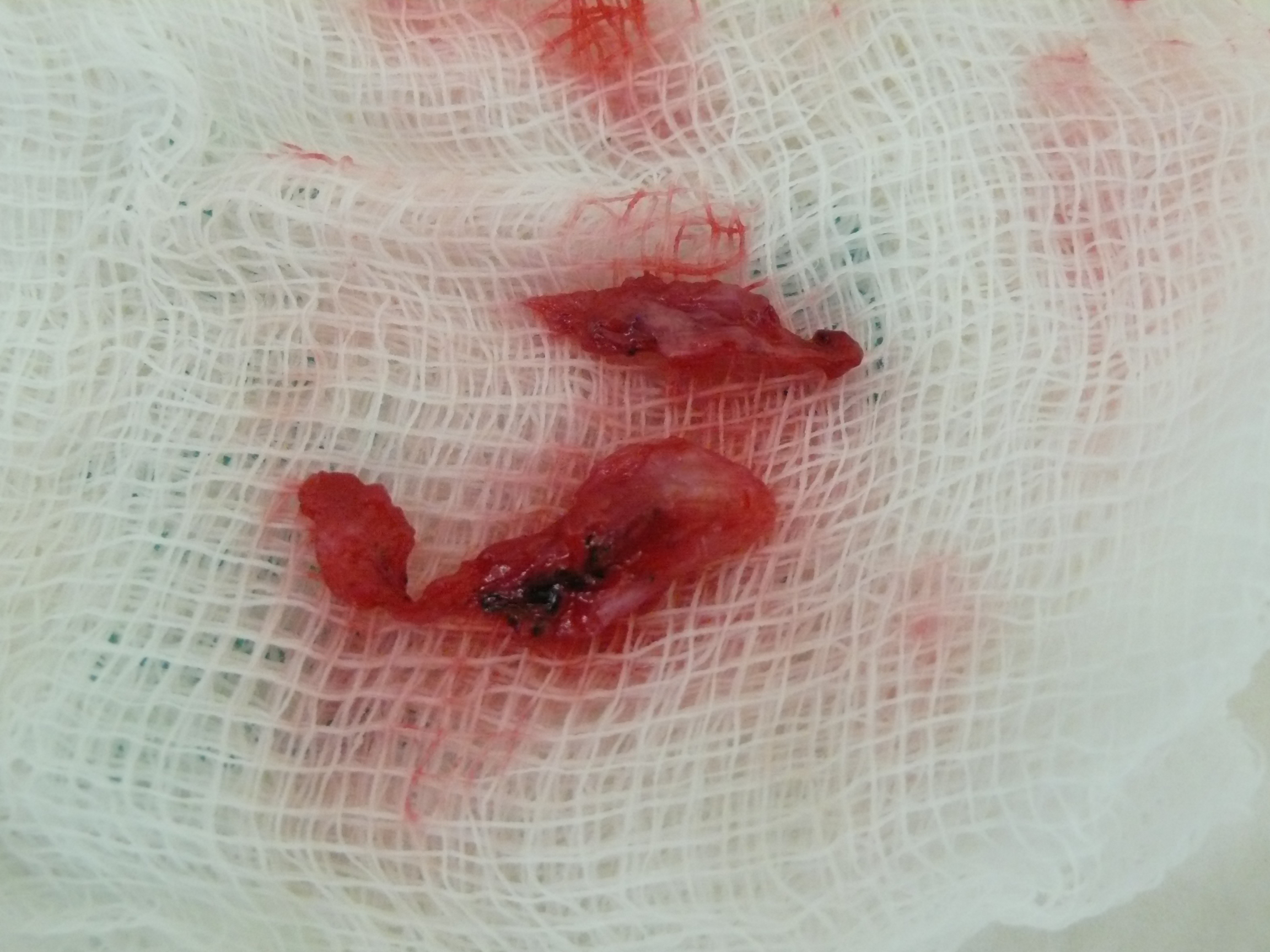

Supplement: Supplementary Image 1 — The gray- pigmented periosteum. [file Image1.JPEG]

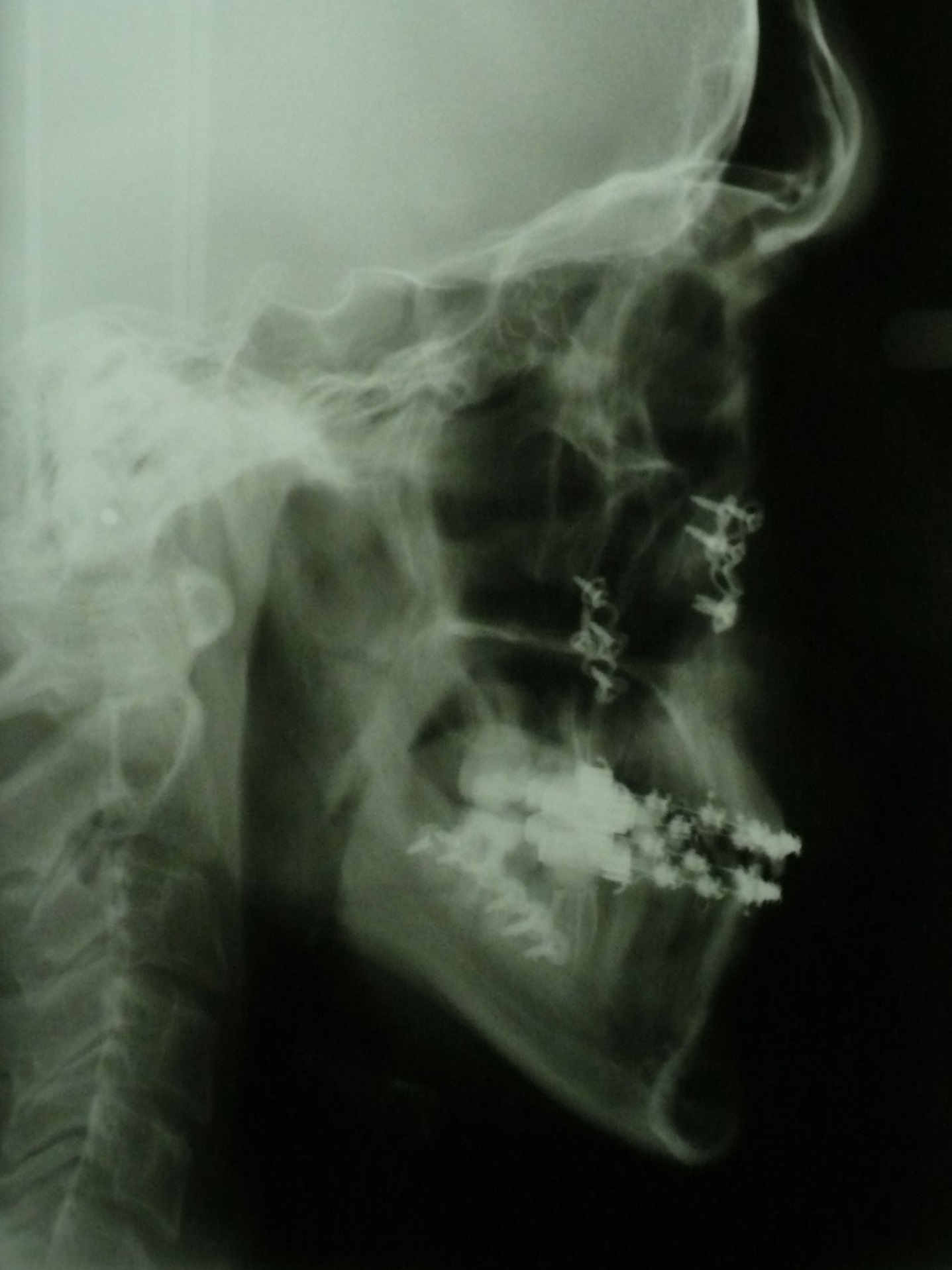

Supplement: Supplementary Image 2 — X ray picture of the patients 23 months after jaw fixation (Siemens Aristos VB20D, 55kV). [file Image2.JPEG]
